# P34 Supporting patients to self-manage infections without antibiotics: views of community pharmacy teams.

**DOI:** 10.1093/jacamr/dlaf118.041

**Published:** 2025-07-14

**Authors:** Aoife Fleming, Iona Nevin-Maguire, Tiarna Sheahan

**Affiliations:** Pharmaceutical Care Research Group, School of Pharmacy, University College Cork, Cork, Ireland; Pharmaceutical Care Research Group, School of Pharmacy, University College Cork, Cork, Ireland; Pharmaceutical Care Research Group, School of Pharmacy, University College Cork, Cork, Ireland

## Abstract

**Background:**

Community pharmacies are often the first port of call for patients seeking clinical advice on minor ailments and infections, with several minor ailment schemes for some infections in pharmacy practice implemented internationally. It is important to consider the views and experiences of community pharmacy teams on managing infections. Through supportive and appropriate self-management advice, patients can manage common self-limiting infections, potentially resulting in reduced antimicrobial prescribing.

**Objectives:**

To explore the views and experiences of community pharmacy staff on their role in supporting patients to self-manage infections.

**Methods:**

Qualitative semi-structured interviews were conducted with community pharmacists and pharmacy staff in November 2022 in Ireland. Participants were purposively sampled from community pharmacies in the south and south-west of Ireland. Participants were invited to share their views and experiences of providing advice on infections, self-management advice, when they would refer patients to a doctor, and their role in AMS. All interviews were conducted on Microsoft Teams and the verbatim transcripts were analysed by thematic analysis. Ethical approval and all participants provided written informed consent.

**Results:**

Interviews were conducted with 19 participants: 14 female; 13 pharmacists, 3 pharmacy counter assistants, 2 pharmacy technicians and 1 pharmaceutical assistant. Participants in detail their approaches to managing minor infections, such as question protocols, self-management strategies, knowledge of warning or referral signs. There were mixed perceptions on the role point-of-care-test services, which are largely not embedded in community pharmacies in Ireland; some suggested they may be beneficial to reduce antibiotic use but could increase workload with an uncertainty around the interpretation of results. Participants highlighted the use of evidence-based information to support their decisions and as information to provide to patients (e.g. paper leaflets, online information). They noted the importance of tailoring this for the individual in terms of health literacy, and how it can empower patients to give them the information to self-manage their infection. Overall participants expressed confidence when providing self-management advice, however they exert extra caution in older persons and children. Recommendations for further training and information, particularly in the area of skin infections and viral infections, were made. Pharmacy teams acknowledged their role in AMS, also noting that they would rarely query an antibiotic prescription from a general practitioner, deferring to the responsibility of prescriber.

**Conclusions:**

This study has provided valuable insights into the views and practices of community pharmacy staff on supporting and advising patients on the self-management of infections without antibiotics. The importance of patient education and evidence-based self-management advice should be considered for future AMS initiatives in community pharmacy practice, to address the objectives of Ireland’s Second One Health National Action Plan.

